# ‘It Just Wears You Down’: A Qualitative Exploration of the Experiences and Wellness Needs of Organ Transplant Caregivers to Inform the Development of Support Resources

**DOI:** 10.3390/healthcare14121679

**Published:** 2026-06-12

**Authors:** Jenna A. P. Sim, Ashley L. Exall, Maneka A. Perinpanayagam, Debra L. Isaac, Kelly W. Burak, Stefan Mustata, S. Nicole Culos-Reed

**Affiliations:** 1 Faculty of Kinesiology, The University of Calgary, 2500 University Drive NW, Calgary, AB T2N 1N4, Canada; jenna.sim@ucalgary.ca; 2 School of Biomedical Engineering, Faculty of Engineering, McMaster University, 1280 Main Street West, Hamilton, ON L8S 4L8, Canada; exalla@mcmaster.ca; 3 Department of Medicine, Cumming School of Medicine, University of Calgary, 3330 Hospital Drive NW, Calgary, AB T2N 2T8, Canada; maneka.perinpanayagam@ucalgary.ca (M.A.P.); dlisaac@ucalgary.ca (D.L.I.); kwburak@ucalgary.ca (K.W.B.); smustata@ucalgary.ca (S.M.); 4 Department of Oncology, Cumming School of Medicine, University of Calgary, 3330 Hospital Drive NW, Calgary, AB T2N 2T8, Canada; 5 Department of Psychosocial Resources, Arthur J.E. Child Comprehensive Cancer Centre, 3395 Hospital Drive NW, Calgary, AB T2N 5G2, Canada

**Keywords:** caregivers, caregiver burden, organ transplantation, family caregivers, caregiver well-being

## Abstract

**Highlights:**

**What are the main findings?**

- Caregivers to organ transplant candidates and recipients experience a high burden of caregiving-related tasks, which can negatively impact their physical and mental well-being.
- Organ transplant caregivers express a need for tailored resources to better support both their caregiving responsibilities and their own health and well-being.

**What are the implications of the main findings?**

- Future work should explore approaches that support patients and caregivers both individually and collectively, considering their interconnected needs and relationship.
- Caregiver needs vary across contexts, relationships, and settings; therefore, interventions should be flexible and tailored to the specific context in which caregiving occurs.

**Abstract:**

Background/Objectives: Caregivers play a critical role in patient care across the pre- and post-transplant periods. However, the demands of caregiving can negatively impact caregivers’ own physical and psychosocial well-being. The Transplant Wellness Program (TWP) is a behavior change intervention that provides exercise support for pre- and post-kidney, pre- and post-liver, and post-lung transplant patients but has not yet included transplant caregivers. Thus, the purpose of this study was to explore the experiences and needs of organ transplant caregivers to inform the development of caregiver-specific support resources for the TWP. Methods: Semi-structured interviews with family caregivers of patients receiving kidney or liver transplant in the TWP were conducted and recorded via Zoom. Interview recordings were transcribed verbatim and analyzed using conventional content analysis. Results: Eight interviews were conducted, with caregivers in both the pre- (*n* = 4) and post-transplant (*n* = 4) periods. Four categories resulted from the data: caregiver strain, life changes, individual wellness needs, and caregiving needs. Nine sub-categories further described caregivers’ experiences and opportunities for wellness support. Conclusions: The caregiving experience was characterized by feelings of overwhelm, stress, and uncertainty. This study highlights the need for comprehensive services such as exercise classes, peer support programs, and tangible aide to support transplant caregivers’ well-being. Three caregiver resources were built out of this study and integrated into the TWP.

## 1. Introduction

Organ transplantation is a lifesaving treatment for end-stage disease and organ failure [1]. However, the transplant journey is complex and taxing on both patients and their family members, as family members often take on a critical caregiving role [2,3,4,5]. In the pre-transplant period, caregivers often assume the majority of household responsibilities such as cooking, cleaning, and grocery shopping and provide home support for the patient’s disease management [2,5,6,7]. At-home disease management support can involve managing and tracking medications and symptoms, at-home treatments such as home hemodialysis or breathing treatments, and scheduling of medical appointments [8,9,10]. The support extends beyond the home as well, with caregivers often attending appointments and acting as the primary source of transportation for the patient [5,8]. Caregivers then face new responsibilities in the post-transplant period as they support patients’ at-home recovery from surgery (e.g., wound care, new medical device management), regain lost strength, and manage new, complex immunosuppressant medication regimens [2,7,11,12].

Caregivers to individuals with chronic diseases experience psychological distress, loss of social opportunities, and reduced physical health and quality of life [4,13,14,15,16,17,18]. Traditional stress and coping models suggest that when an individual perceives a stressor to be present, they will seek out strategies to either directly address the stressor or manage their stress response [19,20]. While there is a growing body of literature identifying the stressors, experiences, and burdens of caregivers to organ transplant patients [3,4,5,7,11], the body of work developing interventions to address the stressors that organ transplant caregivers face remains small [21]. Thus, there remains a gap in understanding how organ transplant caregivers can be best supported to manage the many stressors they face. Across other caregiver populations, physical activity, social support, and self-management interventions have demonstrated success in reducing caregiver distress and burden and increasing overall well-being [22,23,24,25,26,27,28]. However, caregivers’ experiences and needs can vary across contexts; therefore, it is important to engage directly with caregivers to identify their unique needs and develop contextually relevant strategies and interventions to address them.

The Transplant Wellness Program (TWP) is a hybrid effectiveness–implementation trial that delivers exercise behavior change support to kidney, liver, and lung transplant patients in Alberta, Canada [29]. Given the critical role of caregivers, it is important to explore how the TWP can expand to support both patients and caregivers. Thus, the objectives of this study were (a) to describe the experiences and wellness needs of organ transplant candidates and recipients’ caregivers and (b) to develop and integrate resources that address the identified needs within the TWP.

## 2. Materials and Methods

This study was guided by a pragmatic research philosophy, with the aim of learning directly from caregivers to develop practical solutions to support their needs. Reflexivity is a critical component of the pragmatic philosophy; therefore, we feel it is critical to state the positionality of the authors who were directly involved in data collection and analysis. JAPS is a Ph.D. student in kinesiology. Neither she nor her immediate family members had experienced organ transplantation or caring for a transplant patient. JAPS is the daughter of a family caregiver for a patient with advanced dementia and has seen the direct impact that caregiving can have on caregivers’ own well-being. ALE is an undergraduate biomedical engineering student. While she does not have personal experience with organ transplantation or caregiving for transplant patients, ALE has a sister with complex medical needs. This has given her insight into the significant challenges and responsibilities that come with caring for someone with ongoing health issues.

Purposive sampling was used to recruit caregivers via email from the Transplant Wellness Program. Eligible participants were adults who self-identified as the primary caregiver of a transplant patient who was involved with the Transplant Wellness Program. Participants who consented to participate in this study then completed semi-structured interviews over Zoom or telephone. At the time of data collection for this study, the TWP had been recruiting for 6 months and had a relatively small sample of participants. Therefore, to protect individuals’ confidentiality and limit the ability for caregivers’ responses to be linked to TWP participants, it was decided that limited demographic information would be collected. The collected information included the caregiver’s relationship with the patient (parent, spouse, friend, relative), organ type of patient (kidney or liver), and place on the transplant continuum (pre- or post-transplant). The interview guide was semi-structured in nature and included questions regarding caregivers’ role, experiences with caregiving, their self-perceived individual wellness, and engagement in wellness behaviors. Wellness is highly individualized and can look different between persons [30]. As such, participants were first asked about what wellness means to them and the different wellness behaviors they engaged in. If needed, a description of wellness and examples of different wellness behaviors (e.g., sleep, exercise, seeking social support, coping) were provided. For the purpose of this study, wellness was described as the pursuit of and engagement in behaviors and activities that promote an individual’s overall physical, mental, social, and emotional well-being [31]. Participants were then asked about the impacts of caregiving on their individual wellness and ideas of strategies or interventions that would support their wellness.

Interviews were recorded, transcribed verbatim, and inputted into NVivo 12 for analysis by JAPS and ALE. Data were analyzed using conventional content analysis [32], in which transcripts were first read individually by JAPS and ALE to immerse themselves in the data. The first three interviews were then re-read individually, with JAPS and ALE making note of key concepts, impressions, and thoughts as they read the transcripts. An initial meeting was held to discuss the first impressions of the data and develop an initial coding scheme. This initial coding scheme was then used to code the remaining transcripts, with additional codes added when new concepts were identified. A second meeting was then held between JAPS and ALE to identify the links between codes and develop sub-categories and subsequent overarching categories. The senior author (SNC-R) was consulted throughout the analysis period to provide critical feedback and suggestions. To further enhance the rigor of this study, JAPS and ALE engaged in reflexive journaling during the data collection and analysis periods and kept an audit trail to record all decisions.

## 3. Results

**Figure 1:**
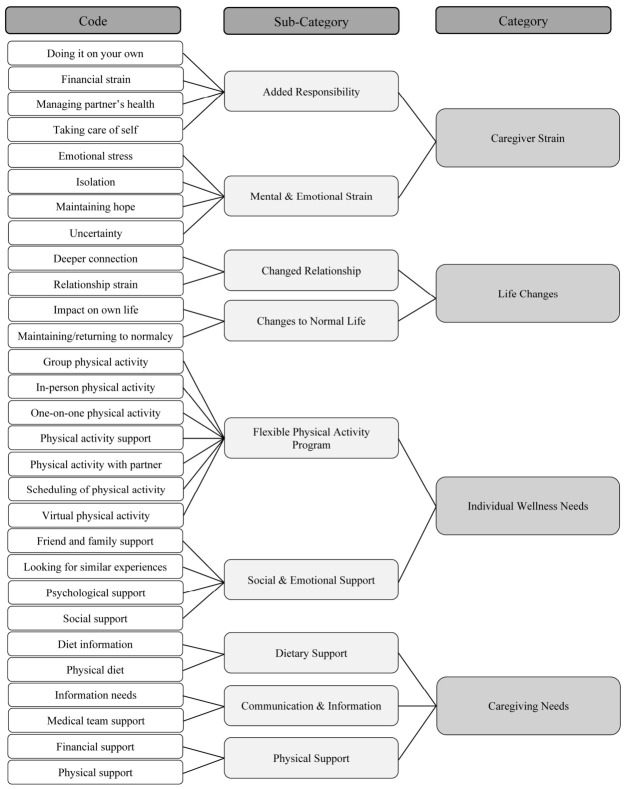
Schematic of codes and categories.

Eleven caregivers were contacted, from which eight participated in the semi-structured interviews. Caregivers included spousal caregivers (*n* = 7), a parental caregiver (*n* = 1) across both kidney (*n* = 6) and liver (*n* = 2) transplant groups. Four caregivers were in the pre-transplant period, and four were post-transplant. Twenty-nine codes were created, which were integrated into nine sub-categories and then four overarching categories (Figure 1).

### 3.1. Caregiver Strain

Participants shared how their caregiving experience was characterized by feelings of overwhelm and stress. Due to their family member’s illness, caregivers had to take on more, often negatively impacting their own well-being.

#### 3.1.1. Added Responsibility

Participants described how they had to take over most household responsibilities and help manage their family member’s health condition. This included activities like ensuring their loved one was meeting their necessary dietary requirements, attending appointments, and supporting dialysis treatments. The strain of additional responsibilities was further exasperated in caregivers who had children or older adult parents to take care of in addition to their family member awaiting transplant.

*“We have this ten-year-old, and we have these two dogs, and you know we have pets, we have a horse, and like all these things need to be taken care of and it’s really just me, right? My wife’s got enough energy to be up and about for an hour, hour and a half a day. So, it all kind of falls on me, you know?”*

*(P3)*

#### 3.1.2. Mental and Emotional Strain

Caring for a family member awaiting transplant was mentally and emotionally stressful for participants. Much of this stress was related to the sense of uncertainty of the transplant journey.

*“You don’t know what’s going to happen. We don’t. You don’t know, is he going to live? Is he not going to live? And if he does get to transplant, what’s it going to be like after”*

*(P1)*

The stress and uncertainty of the pre-transplant period could be unrelenting at times. The impact of this on caregivers’ own well-being was clearly captured by one participant who shared,

*“I can handle a bumpy ride. But it just, it wears on you, you know? It wears you down.”*

*(P3)*

The uncertainty was often coupled with feelings of helplessness. There was little caregivers could do to relieve the physical or emotional pain their family member was experiencing, contributing to caregivers’ own distress.

*“I mean, as a husband, you don’t, you don’t know the fear and the pain or whatever it is that your wife is going through and it’s like there’s nothing you can do for them, right? It feels so helpless. There’s just nothing you can do. Like you can be there and that’s it”.*

*(P7)*

The mental and emotional strain that family caregivers felt was sometimes exacerbated by feelings of isolation. When there were opportunities for social connection, it was still challenging for family caregivers to separate from the mental and emotional strain they were experiencing. During these social interactions, family caregivers worried about potential negative consequences such as bringing home infection or illness. Peers were often unaware of the severity of organ transplant and the strain of caregiving and were unable to empathize.

### 3.2. Life Changes

The category of life changes was characterized by a shift in participants’ identity and responsibilities by assuming the role of a family caregiver. These changes were often unpredictable yet compulsory.

#### 3.2.1. Changed Relationship

Assuming the role of a caregiver led to a significant change in the relationship with their family member. Navigating this ‘new normal’ sometimes led to strain in the relationship between the caregiver and the patient. The inability to engage in activities that the caregiver and patient used to do together at times led to feelings of frustration and resentment about being in this new caregiving position.

*“He doesn’t want to go out anywhere. He doesn’t want to do anything outside…we do our doctor’s visits when we have to, but other than that he doesn’t want to do anything else. Which sometimes annoys me, you know?”*

*(P5)*

The change in familial roles impacted how both the caregiver and patient felt in their relationship. This was more pronounced in spousal relationships where the role change challenged traditional gender roles of being a ‘provider’.

*“If you think that my husband loved it, then you got another story coming to you. There’s no man that wants to be babied by his wife…there’s no man that wants that or no wife that wants that. It’s very, very stressful”*

*(P6)*

While most of the relationship changes led to feelings of stress or friction between the caregiver and their loved one, the parental caregiver expressed that, despite the stress of the transplant, the experience of providing care led to a closer relationship with their adult child. This difference may reflect how the different types of family relationships (e.g., spouse versus parent–child), and the gender roles that are associated in the relationship may influence the caregiving experience.

#### 3.2.2. Changes to Normal Life

In addition to their familial relationship, becoming a caregiver impacted all other areas of life including work, hobbies, and friendships. Participants put off taking on bigger work projects, and some had to switch jobs due to the caregiving demands, contributing to financial stress during this period. If caregivers participated in an activity they did before their family member became sick, they would often feel anxious or guilty for taking time for themselves or were unable to participate to the same extent because of potential risks to their family member. Of interest, one participant noted the difficulty of leaving the caregiver role and returning to ‘normal life’ after their spouse’s transplant,

*“The biggest challenge is just coming back to a place where I can start focusing on my own goals and objectives”*

*(P2)*

Overall, this category was characterized by disruptions to caregivers’ lives and subsequent challenges across domains including relationships, social lives, work, hobbies, and self-identity.

### 3.3. Individual Wellness Needs

Participants discussed the challenge of prioritizing their own wellness while acting as a caregiver. This category captured what participants felt would support their individual wellness while being in a caregiving role.

#### 3.3.1. Flexible Physical Activity Program

Many participants discussed how caregiving negatively impacted their ability to engage in physical activity. There was interest in potential caregiver-specific physical activity programs; however, numerous considerations that would influence their ability to participate in a program were shared. These included scheduling, mode of delivery, level of difficulty, and format. Some participants stated how they thought an in-person program would better support feelings of connection but were concerned of the potential impact on their family member’s condition.

*“[Virtually] you wouldn’t have the interaction there, the back and forth with ideas, for interacting with the peer group. In person would be okay, as long as there’s not big viruses going around. You have immunocompromised people that you don’t want to bring it back to”*

*(P4)*

The option of having a physical activity program being separate from or together with their family member was also discussed. Many participants shared how they thought the option of exercising together with their family member would be beneficial because it would be a break from the medicalization of their relationship and allow them to do something together that they both enjoy. One participant shared how after their family member received the transplant, they were given the option to exercise in the hospital gym together, and this resulted in a continued change in their physical activity.

*“When they started her physio…she had to go to the gym. So, when she went to the gym, I went with her. I signed the waiver, and I started working out with her, which was a great benefit to me…I really enjoyed the time when I got in the gym and even though she’s home [from the hospital], the two of us go to the gym twice a week together”*

*(P8)*

The need and desire for physical activity support was clear amongst caregivers; however, due to the varying abilities of caregivers and patients, instability, and uncertainty associated with transplant, any physical activity program developed must be flexible.

#### 3.3.2. Social and Emotional Support

In addition to caregivers’ physical well-being, additional support for their social, emotional, and well-being is needed. Participants leaned on friends and family for support; however, it was acknowledged that this was often insufficient. Many shared a longing for support from someone else who had been through the transplant caregiving experience before.

*“It would be really nice to be able to have someone to talk to that’s experienced it or gone through it, as opposed to some random psychologist that thinks they can tell you what’s going on. And they’ve never even had a death in their family, let alone someone who is in dire need of a transplant!”*

*(P6)*

### 3.4. Caregiving Needs

The category of caregiving needs represents the needs participants expressed would support them in their ability to provide care to their family member. Three sub-categories within this category represent the distinct areas that more support is needed: dietary support, communication and information, and physical support.

#### 3.4.1. Dietary Support

Caregivers described feeling overwhelmed with the dietary restrictions and guidelines they had to follow. Participants were inundated with rules and foods that their loved one could not eat, which left them feeling stuck and unsure how to move forward. Numerous suggestions for dietary support were brought forward, including a cookbook, menu plans, or meal delivery service.

*“Like if there was like a Hello Fresh [meal kit service] for kidney patients and their families, where, you know, you’re not even preparing the meal, you’re just sending the ingredients to the house for the recipe and saying, “Good luck!” Like that’d be better than trying to plan meals and stuff all the time, you know, like that was that was probably one of the hardest things, trying to come up with meals that fit the diet restrictions, and were still edible”*

*(P2)*

#### 3.4.2. Communication and Information

Participants described the importance of being informed and knowing what to expect with the transplant process. While it was recognized that uncertainty in the transplant journey is unavoidable, participants shared how clear communication positively impacted their caregiving experience.

*“Yes, there was no doubt that if there was any questions to ask, or whatever it was, they were answered, and if they didn’t have the answers they got them for me…everybody was just awesome, that’s all I can say”*

*(P8)*

Caregivers also shared suggestions of what and how information could be shared to better prepare them for the transplant experience.

*“They could have walked through certain scenarios and said, “Okay, if this happens, this is what you’ll will be looking for you to do.” So yeah, maybe something like that, like some different scenarios that could have happened would have been helpful to prepare me a little bit more”*

*(P1)*

#### 3.4.3. Physical Support

Physical support was characterized by services such as housework, cooking, cleaning, driving, and financial support. These were often the tasks that participants had become more responsible for since assuming the caregiver role. Receiving additional help with these tasks was suggested as a way to reduce the overwhelm and strain caregivers felt.

*“Even if you could get like help with groceries, maybe once a month, or gas, like here’s a $200 allowance to help you do something. So, whether that pays a bill, it pays your gas that day, pays your parking for the hospital, pays for me to have my day off. It would be really nice in those scenarios to not have to use a vacation or an unpaid day to go help your significant other for a lifesaving appointment, right?”*

*(P6)*

## 4. Discussion

This study aimed to learn about the experiences and wellness needs of caregivers to organ transplant patients in Alberta to inform the development of caregiver support resources that could be integrated into the TWP. The results from this study align with previous work identifying the negative impact of caregiving on aspects of organ transplant caregivers’ well-being including sleep, quality of life, physical, and mental health [2,4,6,13,15]. However, the qualitative nature of our work can provide additional context and insights into what components of caregiving for transplant patients may negatively influence their well-being. Much of the psychological distress the caregivers in this study experienced was due to the uncertainty of when transplantation would occur and how their family member would fare through the process. While one participant mentioned the support from a healthcare provider in explaining the transplant process and answering questions, this experience was not universal. Most spoke to the need for additional information and communication from both healthcare professionals and peers to be better prepared for their role. The desire for peer support as both a way to gain information and find community was salient across the participants in this study and aligns with the small body of qualitative research with this population, where feelings of isolation, distress, and anxiety have been reported [2,5,6,7,11]. Some work has been conducted to explore the impacts of social support for organ transplant caregivers and found that a Facebook support group can help caregivers to feel more prepared for supporting their loved one through a transplant [2]. As such, building online peer support resources may be important for the TWP and other programs to explore given their low-effort but high-impact potential [2,33,34].

This study also highlights the impacts of caregiving on organ transplant caregivers’ physical health and physical activity behavior. Many shared losing their main physical activity partner and having to re-allocate personal time for caregiving duties once their loved one became sick, resulting in a decrease in physical activity. Given the well-established benefits of physical activity on caregivers’ well-being [26], it will be important for future work to explore how to best support organ transplant caregivers to engage in physical activity. The next steps could include the development of patient–caregiver dyadic interventions, as they have demonstrated success in other chronic disease populations [35,36,37]. Beyond their personal well-being, the caregivers in this study also identified the need for more support to carry out their caregiving duties. Of interest, while the interview guide used in this study did not include any diet-specific questions, nearly all caregivers described difficulty in managing patients’ dietary needs. This aligns with previous work in both the transplant patient and caregiver literature, documenting the challenges of managing transplant-related dietary restrictions [11,38,39]. There is a clear need to further explore how to best support transplant patients and caregivers in managing their dietary restrictions.

The use of theories, models, and frameworks can help to further understand potential reasons why and identify next steps in addressing the aforementioned needs of organ transplant caregivers. Lazarus and Folkman’s transactional theory of stress and coping suggests that when individuals determine a stressor to be present, they engage in coping strategies to either manage their response to the stressor or to directly address the stressor itself, after which, individuals re-assess the situation. If the stressor is still producing distress or negative emotions, they may engage in additional coping strategies to try and address the stressor [19,20]. This study identified that while organ transplant caregivers acknowledge the stressors present (uncertainty of transplant, additional responsibilities, dietary restrictions), many struggled to be able to engage in coping behaviors (seeking social support, physical activity) that would help to manage the stressor. Behavior change theories can offer further insight as to why transplant caregivers experience this gap between acknowledgement of the stressor and engagement in the coping behavior. For example, the capability, opportunity, motivation and behavior (COM-B) model suggests that an individual’s engagement in a behavior is dictated by their capability (physical and psychological), opportunity (physical and social), and motivation (reflective and automatic) [40]. Most participants in this study expressed a desire for coping resources such as more information, social support, and physical activity opportunities but were unaware if resources existed. This suggests that organ transplant caregivers are motivated to engage in coping behaviors but lack the opportunity to do so. Therefore, future work may consider developing resources such as peer support networks, physical activity interventions, or education sessions to increase caregivers’ opportunity to engage in coping behaviors. In addition, this study highlights how the stressors that caregivers face can change across the transplant continuum (i.e., uncertainty pre-transplant, returning to ‘normal’ post-transplant), as well as how differing relationships (i.e., parent vs. spouse) can impact how the caregiver appraises the stressor. Given the small and descriptive nature of this study, it will be important for future work to explore how to tailor resources to support differing caregiver–patient relationships and timepoints on the transplant continuum. In building resources that address these factors, organ transplant caregivers may be supported to better cope with the numerous stressors they encounter in their role.

### 4.1. Development of TWP Caregiver Resources

Results from this work have informed the development of several caregiver support resources within the TWP. After analysis was completed, planning meetings were held between the first (JAPS), third (MAP), and senior (SNC-R) authors to determine feasible next steps to address identified needs. The categories of Individual Wellness Needs, and Caregiving Needs and subsequent sub-categories guided the development of the caregiver support resources. Due to feasibility, it was determined that any support resources would need to be integrated within the existing TWP structure or be available as open-access online resources. The primary focus of the TWP is exercise behavior change and includes bi-weekly exercise classes, one-on-one exercise counselling, and monthly ‘wellness webinars’ covering topics such as self-compassion, goal setting, and fatigue management. To address the need for dietary support, nutrition webinars delivered by registered dietitians at the local kidney disease clinic are now offered bi-monthly as part of the ‘wellness webinars’ component of the intervention and are open to both patients and caregivers. In the planning meetings, it was determined that delivering caregiver-specific exercise classes was not feasible for the TWP. Rather, caregivers are now invited to participate alongside their loved one in most components of the TWP. At intake to the TWP, participants are asked about their caregiver or support person. If the patient identifies a primary caregiver, they are informed that the caregiver is welcome to join the exercise counselling calls, group wellness webinars, and online exercise classes. At this time, only caregivers who reside at the same address as the patient can join the exercise classes due to study-established emergency protocols. Finally, to address the social and emotional support as well as more communication and accessible information needs, a caregiver testimonial video series was developed. Three caregivers, one post-kidney transplant and two pre-kidney transplant, were interviewed to share their experiences, lessons learned, and advice for other caregivers. This series is publicly available on YouTube and all participants in the TWP are sent a link to the series to share with their support network. While the TWP was unable to address all needs identified in the present study, these adaptations represent realistic and feasible steps for the TWP to support caregivers while maintaining its primary focus on patient well-being.

### 4.2. Strengths and Limitations

This study is limited in scope given its focus on the context of the TWP, southern Alberta, and small sample size. While a large sample size is not a requirement of qualitative research [41], the experiences described in this study represent a small and context-specific group of organ transplant candidate and recipient caregivers. Thus, the results may not be generalizable to organ transplant caregivers across all contexts. As well, only kidney and liver transplant patient caregivers are represented in this study. At the time of data collection, the TWP had not yet expanded to include lung transplant patients. Therefore, the experiences of these caregivers are not represented in this study. As such, future work should explore the needs of heart, lung, and multi-organ transplant caregivers. The strengths of this study include its’ pragmatic focus; the results of this study were translated into tangible resources for caregivers in the TWP. Including perspectives from both pre- and post-transplant caregivers provided insight into how caregiver needs change across the transplant continuum, highlighting the importance of developing resources that address differing needs at multiple time points to ensure caregivers are adequately supported.

## 5. Conclusions

This study identified that caregivers to patients receiving organ transplants in southern Alberta experience strain on all aspects of their well-being due to the isolation, uncertainty, and disruption that transplantation causes. Caregivers require more resources to both fulfill their caregiving duties and prioritize their own health and well-being. While the TWP has developed some resources to support caregivers’ well-being and their ability to support the transplant patient, there remains additional gaps that warrant further exploration, such as caregiver-specific support groups, peer counseling, and dyadic interventions. Caregivers are a critical part of the organ transplant care team; thus, continuing to gather information and co-develop support resources that will meet their needs is a critical next step.

## Data Availability

The data presented in this study may be available on request from the corresponding author; the data are not publicly available due to ethical restrictions.

## Abbreviations

The following abbreviations are used in this manuscript:

TWP	Transplant Wellness Program
